# Metabolic profiling of maternal serum of women at high-risk of spontaneous preterm birth using NMR and MGWAS approach

**DOI:** 10.1042/BSR20210759

**Published:** 2021-08-31

**Authors:** Juhi K. Gupta, Angharad Care, Laura Goodfellow, Zarko Alfirevic, Lu-Yun Lian, Bertram Müller-Myhsok, Ana Alfirevic, Marie M. Phelan

**Affiliations:** 1 Wolfson Centre for Personalised Medicine, Department of Pharmacology and Therapeutics, Institute of Systems, Molecular and Integrative Biology, University of Liverpool, Liverpool, L69 3GL, UK; 2 Harris-Wellbeing Research Centre, University Department, Liverpool Women’s Hospital, Liverpool, L8 7SS, UK; 3 NMR Centre for Structural Biology, Institute of Systems, Molecular and Integrative Biology, University of Liverpool, Liverpool, L69 7ZB, UK; 4 Max Planck Institute of Psychiatry, Munich 80804, Germany

**Keywords:** biomarker discovery, metabolomics, mGWAS, multiple omics, NMR, Preterm birth

## Abstract

Preterm birth (PTB) is a leading global cause of infant mortality. Risk factors include genetics, lifestyle choices and infection. Understanding the mechanism of PTB could aid the development of novel approaches to prevent PTB. This study aimed to investigate the metabolic biomarkers of PTB in early pregnancy and the association of significant metabolites with participant genotypes. Maternal sera collected at 16 and 20 weeks of gestation, from women who previously experienced PTB (high-risk) and women who did not (low-risk controls), were analysed using ^1^H nuclear magnetic resonance (NMR) metabolomics and genome-wide screening microarray. ANOVA and probabilistic neural network (PNN) modelling were performed on the spectral bins. Metabolomics genome-wide association (MGWAS) of the spectral bins and genotype data from the same participants was applied to determine potential metabolite-gene pathways. Phenylalanine, acetate and lactate metabolite differences between PTB cases and controls were obtained by ANOVA and PNN showed strong prediction at week 20 (AUC = 0.89). MGWAS identified several metabolite bins with strong genetic associations. *Cis*-eQTL analysis highlighted *TRAF1* (involved in the inflammatory pathway) local to a non-coding SNP associated with lactate at week 20 of gestation. MGWAS of a well-defined cohort of participants highlighted a lactate-*TRAF1* relationship that could potentially contribute to PTB.

## Introduction

Preterm birth (PTB) is defined by the World Health Organisation as birth prior to 37 weeks of gestation [1]. PTB complications are the leading cause of death in children under the age of five [2], but this multifactorial condition is not fully understood. Environmental factors are known to influence PTB and include nutrition, maternal stress or infection [1]. Metabolomics is a field that monitors global metabolism of a system, and this can be influenced by both environmental and genetic factors [3,4]. Clinical phenotypes of non-iatrogenic spontaneous labour include spontaneous preterm birth (SPTB) and PPROM (preterm premature rupture of membranes). To provide targeted SPTB preventative therapies, reliable biomarkers of women with pregnancies destined to labour prematurely are required. This can be achieved by understanding the pathophysiology behind the initiation of early labour. Different regulation axis between SPTB and PPROM have previously been suggested [5].

Many metabolomics studies applied less than 37 weeks of gestation as a cut-off for PTB [6,7] yet there is an inverse relationship between gestation at birth and morbidity and mortality on the infant. Fortunately, 70% of preterm births are late preterm births (34–36^+6^) and have a low burden of morbidity [8]. However, this makes it difficult to study the preterm pregnancies most in need of prevention. Therefore, in the present study we obtained cleaner phenotypes of PTB cases and defined outcomes as ≤ 34 weeks, in line with preterm birth prevention services in the UK [9].

Recent PTB metabolomics studies [6,10,11] have utilised non-targeted analytical techniques such as nuclear magnetic resonance (NMR) to identify biomarkers in systemic fluids: cervicovaginal fluid, blood plasma and urine respectively. Graca et al. [12], who applied mass-spectrometry, and Amabebe et al. [6] proposed several metabolite predictors for PTB including acetate, lactate, phenylalanine and other amino acids. NMR is a highly sensitive and reproducible method with high-throughput automated sample processing, which is advantageous for large sample size studies screening biofluids [13–16].

Probabilistic neural network (PNN), a supervised machine learning method, can accurately classify metabolomic data with respect to phenotype [17,18]. Rueedi et al. [19] applied metabolomics genome-wide association (MGWAS) method to gain an understanding of interactions between single-nucleotide polymorphisms (SNPs) and the metabolome in serum samples. As metabolites are phenotype-driven, associating metabolites with genome-wide data could enhance our knowledge of the pathways these metabolites are involved in. These, in addition to PNN, are novel approaches to understanding the mechanism of early labour.

Metabolic profiling to distinguish the different clinical subtypes of PTB, preterm premature rupture of the membranes (PPROM) and spontaneous preterm birth (SPTB) has not yet been reported. A non-invasive test of systematic fluids could help screen and diagnose women susceptible to PTB in early pregnancy allowing for closer monitoring and preventative treatment. This 'omics’ approach can also improve sensitivity and specificity of biomarkers, compared with traditional clinical markers, by screening a population for multiple metabolites as reviewed by Monteiro et al. [20].

The present study aimed to investigate the serum metabolome from a unique cohort of high-risk PTB women in early pregnancy using untargeted 1-dimensional ^1^H (proton) NMR. An MGWAS approach was subsequently applied to investigate genetic contributions from the most promising metabolites with the aim to identify underlying pathways of early labour.

## Materials and methods

### Participant recruitment

Participants with singleton pregnancies, visiting between April 2012 to December 2017, were prospectively recruited at 16 and 20 weeks of gestation at the Liverpool Women’s Hospital Preterm Birth Prevention Clinic. Research ethics approval was obtained from the North West Research Ethics Committee, (REC reference: 11/NW/0720) and informed consent was obtained from the participants. Clinical data on recruitment and delivery outcomes were collected and followed up in this nested case–control study. Women who previously experienced preterm birth (≤ 34 weeks) and subsequently delivered ≤ 37 weeks in the index pregnancy were categorised as high-risk (HTERM). Low-risk control patients were parous women with all previous births at term and who delivered ≥ 39 weeks in the index pregnancy (LTERM). Women who had spontaneous PTB ≤ 34^+0^ weeks gestation were reviewed and classified as a phenotype of SPTB or PPROM. All births were phenotyped by authors A.C. and L.G., any disagreement in classification was resolved by Z.A.

Participants were excluded if (i) they had a caregiver initiated preterm birth for other pregnancy specific pathology (e.g. pre-eclampsia), (ii) intrauterine death occurred, (iii) had multiple pregnancies, (iv) underwent iatrogenic PTB and (v) their spontaneous PTB occurred ≥ 34^+0^ weeks. Women from the low-risk control cohort were included if they delivered ≥ 39 weeks (and excluded if they delivered < 39 weeks or received PTB prevention treatment [progesterone, cervical pessary or cervical cerclage]). There is now ample evidence that many infants born at 38 weeks of gestation or less experience an increase in neonatal mortality and even lifetime morbidity related to immaturity of one or more organs when compared with infants born at 39 weeks or greater [21–25]. The arbitrary definition of a healthy term birth being anything at or greater than 37 weeks does not correspond with functional maturity and as such may make this definition redundant. For this reason, we, in agreement with others [26] believe defining term births as those occurring at 39 weeks more appropriate.

This article will hereon refer to all non-iatrogenic spontaneous PTB as sPTB (the sub-categories of which are SPTB and PPROM).

### Sample collection

Samples were collected from all women who attended the clinic at 16 and 20 weeks of gestation. In addition, women who only attended the clinic at either 16 or 20 weeks of gestation were included (7.8% of all women sampled). Maternal serum samples were collected in BD vacutainer® with clot activators and stored at room temperature (20– 25°C) for 30 min. The samples were centrifuged at 3000 rpm at 4°C for 10 min. Aliquots of 500 µl were prepared and stored at −80°C.

### Sample preparation and NMR acquisition

Serum (500 µl) was thawed at room temperature and diluted with 500 µl of 200 mM phosphate (PO_4_^3−^) buffer (pH 7.4) and deuterated water. Phosphate buffer was made using dibasic sodium phosphate (Na_2_HPO_4_, VWR International, US: Mr = 141.96) and monobasic sodium phosphate (NaH_2_PO_4_, Acros Organics Fisher Scientific, UK: Mr = 119.98) in 20% ^2^H_2_O (Sigma, UK). In approximately 4.5% of samples, serum volume available was <500 µl, 200 µl of ddH_2_O was added to ensure a volume of >500 µl diluted serum (all samples were normalised including those diluted to mitigate batch effects and the diluted samples were spread equitably between all four sample groups, ranging between 2.4 and 5.6%). Samples were briefly vortexed and centrifuged at 21.5 rpm (∼21,500 ***g***), 4°C for 5 min. Serum-buffer mix (600 µl) was transferred into 5 mm diameter NMR tubes for processing on the 600 MHz NMR solution-state spectrometer Bruker Avance III system (Bruker, GmBH, Germany). A one-dimensional vendor supplied (1D) Carr-Purcell-Meiboom-Gill (CPMG) pulse sequence was applied to attenuate signals from high molecular weight components [27]. Spectra were acquired at 37°C, with 32 transients, 20 ppm spectral width with 4s interscan delay – full parameter sets are available with the deposited dataset (MTBLS1990). Spectra were automatically processed via standard vendor routines to ensure consistent Fourier transformation, window function and phasing (Bruker macro apk0.noe). Spectra were aligned indirectly to Trimethylsilyl propionate via glucose anomeric doublet (5.204 ppm). Quality control (QC) measures were performed manually according to 'The Metabolite Standards Initiative' – MSI [28] briefly via measurement of the Line-Width Half Height (LWHH) of a representative peak – the anomeric glucose located 5.244 ppm (< 3 Hz), ensuring a flat baseline for each sample spectrum, checking consistent signal-to-noise ratio across spectra and ensuring good water suppression (i.e. water signal is narrow, and < 0.4 ppm wide). Where the spectra failed QC the sample were repeated.

### Metabolite annotation and identification

Metabolite annotation was determined through peak fitting of serum spectra initially using the metabolite library provided through Chenomx software v8.2 (Chenomx Inc., Canada) followed by comparison to in-house library spectra for specific metabolites of interest (using 1D ^1^H and 2D ^1^H ^13^C HSQC where appropriate standard spectra acquired on the same instruments under identical conditions). Peaks were converted to spectra 'bins' by manual preparation of a ’pattern file' (a text file generated manually defining the left and right ppm boundaries for each individual peak [or multiplet] in the spectrum). Peaks were then integrated over each bin boundary and divided by the width of each bin using the software AMIX (Bruker, Coventry U.K.). The resultant bin or bucket table contained 145 individual bins, 99 with metabolite specific annotation corresponding to 34 unique metabolites. A further 46 bins that were not assigned to known metabolite signals. Metabolite annotations and identifications are defined as per 'The Metabolite Standards Initiative' – MSI [28], briefly metabolites annotated to an external library (i.e. Chenomx) were assigned to 'level 2 - annotation' whereas metabolites with identities confirmed using two independent terms (such as ^1^H and ^13^C chemical shifts) from an in-house source were assigned to 'level 1 - identification'. Representative spectra can be found in Supplementary Figure S1. Peak boundaries (pattern file) associated annotations and raw data are accessible via open access repository MetaboLights [29], study ID: MTBLS1990.

### NMR spectral bins analysis

Univariate statistical analysis was performed using R statistical computing environment [r-project.org]. Data were first normalised per spectrum using Probabilistic Quotient Normalisation (PQN) [30] to offset any dilution effects from the sample preparation. One-way ANOVA with Tukey’s HSD multiple testing was employed on the 145 variable dataset using R packages 'car' (Companion to Applied Regression). The four individual phenotypes (HTERM, LTERM, SPTB and PPROM) were included in the analysis and a significance level of *P* < 0.05 was applied [31]. MetaboAnalyst was used to perform multivariate analysis with spectra normalised to the median and variables scaled using the Pareto method. Principal component analysis (PCA) was employed to appraise spectra quality and ensure no outliers were present and partial least squares discriminant analysis (PLS-DA) a widely used supervised, multivariate, classification method in chemometrics. PLS-DA classification, with 10-fold cross-validation to evaluate the predictive model, using R packages 'pls’ [32] and ’caret' [33]. The *Q*^2^ value describes the cross-validated sum of squares (*R*^2^), which is provided with the model prediction accuracy score by MetaboAnalyst [34]. The PNN algorithm was executed on all 145 metabolite bins per timepoint using a predictive modelling software: DTREG (https://www.dtreg.com/) [35]. Leave-one-out cross-validation (LOOCV) was applied and the AUC values obtained for each gestation timepoint. For this analysis, the phenotypes were combined: sPTB cases (SPTB and PPROM) and controls (HTERM and LTERM).

### DNA preparation and genome-wide screening

DNA was extracted from whole blood using the Chemagenic Magnetic Separation Module I (Auto Q Biosciences Ltd, U.K.) and were processed on the Applied Biosystems™ UK Biobank Axiom™ array (Thermo Fisher Scientific) for genome-wide screening by the Oxford Genomics Centre at the Wellcome Centre for Human Genetics.

### Genome-wide association (GWAS) data quality control and imputation

Genotypes acquired from the UK Biobank Axiom™ array (Thermo Fisher) were analysed using PLINK v1.9 software [36]. Data quality control (QC) was carried out using the methods described by Anderson et al. [37] and Marees et al. [38]. QC steps involved removing SNPs with low genotype call rate, or high proportion of SNP missingness; checking for gender discrepancies based on heterozygosity in chromosome X; excluding SNPs with low minor allele frequency (MAF) of < 1% and removing SNPs deviating from Hardy–Weinberg Equilibrium (HWE) at *P* ≤ 1 × 10^−6^. Samples with high or low heterozygosity rates (± 3 standard deviations from the mean) or close relatedness (pi-hat score > 0.2) were excluded. Individuals not genetically assigned to European ancestry (CEU) population based on the HapMap data were also excluded from the study [39,40]. A total of 618,283 SNPs were uploaded on to the Michigan Imputation Server for phasing chromosome 1 to 22 using Eagle v2.3 and imputation (using the minimac3 algorithm) against HRC r1.1 2016 panel [41,42]. Post-imputation QC steps involved removing variants with *R*^2^ < 0.3 [43] and MAF = 0 (or < 1%).

### MGWAS and SNP annotation

Inverse-rank normalisation was applied to all the metabolite bin relative peak abundances. Frequentist association test of the GWAS SNP data and the NMR metabolite bins as a continuous outcome was completed using SNPTEST v2.5 [44–46]. Manhattan plots were generated using qqman R package [47]. Total number of samples included at week 16, *n*=251 and at week 20, *n*=265.

SNPs above suggestive threshold of 1 × 10^−5^ were selected from each MGWAS analysis and further investigated for functional annotation of the SNPs and expression quantitative trait locus (eQTL) FUMA GWAS (Functional Mapping and Annotation of Genome-Wide Association Studies) SNP2GENE [48].

### eQTL mapping and enrichment analysis

Lead SNPs were defined as *P* ≤ 1 × 10^−5^ using 1000Genomes phase 3 European population [49,50] as the reference panel, GTEx v8 database tissue types for eQTL mapping and eQTL FDR *P* < 0.05 cut-off. For gene set enrichment analysis and annotation in biological context, SNP2GENE results of genome-wide significant SNPs were submitted to FUMA GENE2FUNC, using GTEx v8 tissue types.

## Results

### Participants

**Figure 1:**
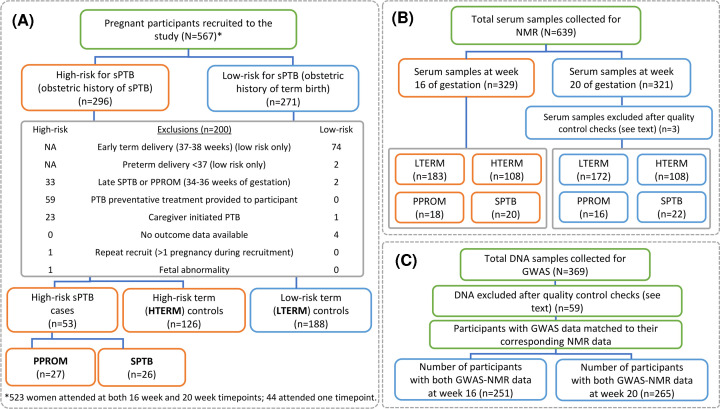
Schematic of pregnant participants recruited to the Liverpool preterm birth study cohort and the number of samples acquired (**A**) Final number of women for each phenotypic group included in the analyses. (**B**) Serum samples were collected from participants at 16 and/or 20 weeks of gestation (of those included in the study analyses) for metabolomics. (**C**) Whole blood-extracted DNA was collected for genotyping (for women included in the study analyses); GWAS, genome-wide association study; HTERM, high-risk term births; LTERM, low-risk term births; NMR, nuclear magnetic resonance; PPROM, preterm premature rupture of membranes; sPTB, spontaneous preterm birth (including PPROM and SPTB); SPTB, spontaneous preterm birth.

A total of 567 women were recruited and categorised into delivery phenotypes or excluded from the study (Figure 1A).

Table 1 summarises the participant demographics included in metabolomics analyses, after three samples were excluded at week 20, due to EDTA contamination (Figure 1B). Recruits missing visits at either timepoint remained in the analyses as a single sample.

**Table 1:** Baseline demographics of PTB metabolomics participants at week 16 and 20 of gestation

	Week 16 of gestation					Week 20 of gestation				
	LTERM (*N*=183)	HTERM (*N*=108)	PPROM (*N*=18)	SPTB (*N*=20)	*P* value	LTERM (*N*=172)	HTERM (*N*=108)	PPROM (*N*=16)	SPTB (*N*=22)	*P* value
Age					**0.837** ^1^					**0.750** ^1^
Mean (SD)	31.0 (4.7)	30.5 (5.1)	30.8 (4.9)	30.9 (6.4)		31.2 (4.6)	30.8 (5.2)	29.9 (4.8)	30.8 (5.9)	
BMI					**0.393** ^2^					**0.394** ^2^
Median (Range)	24.0 (17.0, 57.0)	25.0 (18.0, 43.0)	25.0 (17.0, 39.0)	28.0 (17.0, 49.0)		24.0 (17.0, 57.0)	25.0 (18.0, 43.0)	25.0 (17.0, 39.0)	27.5 (17.0, 49.0)	
Smoking					**0.009** ^3^					**0.095** ^3^
NA	2 (1.1%)	1 (0.9%)	1 (5.6%)	0 (0.0%)		2 (1.2%)	1 (0.9%)	0 (0.0%)	0 (0.0%)	
No	163 (89.1%)	82 (75.9%)	12 (66.7%)	16 (80.0%)		154 (89.5%)	85 (78.7%)	12 (75.0%)	18 (81.8%)	
Yes	18 (9.8%)	25 (23.1%)	5 (27.8%)	4 (20.0%)		16 (9.3%)	22 (20.4%)	4 (25.0%)	4 (18.2%)	
Ethnicity					**0.251** ^3^					**0.150** ^3^
White	177 (96.7%)	99 (91.7%)	17 (94.4%)	20 (100.0%)		166 (96.5%)	99 (91.7%)	14 (87.5%)	22 (100.0%)	
Black	3 (1.6%)	8 (7.4%)	1 (5.6%)	0 (0.0%)		3 (1.7%)	7 (6.5%)	1 (6.2%)	0 (0.0%)	
Other	2 (1.1%)	0 (0.0%)	0 (0.0%)	0 (0.0%)		2 (1.2%)	0 (0.0%)	0 (0.0%)	0 (0.0%)	
NA	1 (0.5%)	1 (0.9%)	0 (0.0%)	0 (0.0%)		1 (0.6%)	2 (1.9%)	1 (6.2%)	0 (0.0%)	
No. of prior SPTB/PPROM <34 weeks										
0	183 (100.0%)	0 (0.0%)	0 (0.0%)	0 (0.0%)		172 (100.0%)	0 (0.0%)	0 (0.0%)	0 (0.0%)	
1	0 (0.0%)	102 (94.4%)	13 (72.2%)	14 (70.0%)		0 (0.0%)	102 (94.4%)	12 (75.0%)	16 (72.7%)	
2	0 (0.0%)	6 (5.6%)	4 (22.2%)	5 (25.0%)		0 (0.0%)	6 (5.6%)	3 (18.8%)	5 (22.7%)	
3	0 (0.0%)	0 (0.0%)	0 (0.0%)	0 (0.0%)		0 (0.0%)	0 (0.0%)	0 (0.0%)	0 (0.0%)	
4	0 (0.0%)	0 (0.0%)	1 (5.6%)	1 (5.0%)		0 (0.0%)	0 (0.0%)	1 (6.2%)	1 (4.5%)	
Previous significant cervical surgery					**0.032** ^3^					**0.135** ^3^
No	180 (98.4%)	108 (100.0%)	16 (88.9%)	20 (100.0%)		169 (98.3%)	108 (100.0%)	15 (93.8%)	22 (100.0%)	
Yes^4^	3 (1.6%)	0 (0.0%)	2 (11.1%)	0 (0.0%)		3 (1.7%)	0 (0.0%)	1 (6.2%)	0 (0.0%)	
Gestation at sampling (days)					**<0.001** ^2^					**<0.001** ^2^
Median (IQR)	117.0 (5)	114.0 (5)	113.5 (5)	113.5 (5.2)		144.5 (5)	142.0 (5)	143.5 (6.5)	143.0 (3.8)	

Participant serum samples collected at these timepoints, included in the analyses, are shown in these final numbers.

1 Linear Model ANOVA.

2 Kruskal–Wallis rank sum test.

3 Fisher’s Exact Test for Count Data.

4 Previous large loop excision of the transformation zone (LLETZ), multiple LLETZ or Knife Cone Biopsy.

### Metabolomics findings

ANOVA, with Tukey’s HSD of the four individual phenotypes, showed 22 metabolite bins at week 16 to be significant (*P* < 0.05) mainly between the control groups LTERM and HTERM (Table 2). Four metabolite bins were significant (Tukey’s post-hoc *P* < 0.05) for SPTB-LTERM comparison: these were unknown (3.32 ppm), unknown (7.28 ppm), unknown (4.40 ppm) and glucarate (4.14 ppm). When comparing PPROM-LTERM, only three bins were significant (*P* < 0.05): unknown (3.32 ppm), unknown (3.28 ppm) and unknown (3.30 ppm).

**Table 2:** Summary of 22 significant metabolite bins (*P*<0.05) at week 16 of gestation shown by ANOVA with Tukey’s HSD

Metabolite bin (chemical shift in ppm)	*P*-value	Tukey’s HSD	*P*-adjusted
Unknown (3.32)	1.90E-07	LTERM-HTERM	2.67E-05
		SPTB-LTERM	0.002
		PPROM-LTERM	0.003
Unknown (3.28)	2.30E-04	PPROM-LTERM	0.010
		LTERM-HTERM	0.012
Unknown (7.28)	4.88E-04	SPTB-LTERM	0.003
Unknown (3.3)	0.001	PPROM-LTERM	0.013
		LTERM-HTERM	0.026
Unknown (4.40)	0.005	SPTB-LTERM	0.019
Glucarate (4.14)	0.005	SPTB-LTERM	0.011
Phenylalanine (7.43)	0.005	NA	NA
Tyrosine (6.90)	0.006	LTERM-HTERM	0.020
Creatine40 (3.93)	0.01	LTERM-HTERM	0.013
Unknown (1.92)	0.011	LTERM-HTERM	0.010
Acetate (1.92)	0.011	LTERM-HTERM	0.009
Unknown (3.92)	0.013	LTERM-HTERM	0.010
Glucose (3.91)	0.014	LTERM-HTERM	0.007
Choline (3.19)	0.019	LTERM-HTERM	0.020
NDMA (3.15)	0.022	LTERM-HTERM	0.021
Glucose (3.52)	0.027	LTERM-HTERM	0.015
Mobile lipids (1.23)	0.028	LTERM-HTERM	0.035
2-Hydroxybutyrate (4.02)	0.039	NA	NA
Glucose (3.78)	0.043	LTERM-HTERM	0.027
Unknown (3.63)	0.044	LTERM-HTERM	0.033
Unknown (3.56)	0.048	NA	NA

A total of 12 bins were annotated metabolites and 10 were unknown. HTERM (*n*=108), LTERM (*n*=183), PPROM (*n*=18) and SPTB (*n*= 20).

NA = the individual outcome comparison did not meet the *P*≤0.05 cut-off.

ANOVA of week 20 metabolite bins highlighted 34 significant bins (Tukey’s post-hoc *P* < 0.05), 12 more than week 16 (Table 3). SPTB-LTERM showed 15 metabolite bins were significant at *P* < 0.05, 11 bins for PPROM-LTERM and 1 bin (unknown [7.28 ppm], Tukey’s post-hoc *P* = 0.036) for SPTB-HTERM. Unknown (3.32 ppm) and unknown (3.28 ppm) were also significant in week 20 similarly to week 16, warranting further investigation. More annotated metabolites were identified at week 20, such as 2-hydroxybutyrate (4.02 ppm) (*P* = 2.16E-05) and creatinine (4.06 ppm) (*P* = 1.63E-04). More LTERM and SPTB/PPROM comparisons were significant (*P* < 0.05), including lactate and glucarate (4.13 ppm), lactate (4.11 ppm) and lactate (1.33 ppm).

**Table 3:** Summary of 34 significant metabolite bins at week 20 of gestation with *P*<0.05 ANOVA with Tukey’s HSD

Metabolite bin (chemical shift in ppm)	Annotation level (MSI)	*P*-value	Tukey’s HSD	*P*-adjusted
Unknown (3.32)	4	4.77E-08	LTERM-HTERM	7.27E-05
			PPROM-LTERM	1.20E-04
			SPTB-LTERM	0.002
2-Hydroxybutyrate (4.02)	2	2.16E-05	PPROM-LTERM	0.001
			LTERM-HTERM	0.007
			SPTB-LTERM	0.024
Unknown (7.28)	4	5.12E-05	SPTB-LTERM	1.15E-04
			LTERM-HTERM	0.025
			SPTB-HTERM	0.036
Creatinine (4.06)	1	1.63E-04	SPTB-LTERM	0.002
			PPROM-LTERM	0.017
Glucarate and myoinositol (4.04)	2 and 1	2.51E-04	PPROM-LTERM	0.012
			SPTB-LTERM	0.022
			LTERM-HTERM	0.022
Unknown (3.28)	4	0.003	PPROM-LTERM	0.011
Lactate and glucarate (4.13)	1 and 2	0.004	PPROM-LTERM	0.033
			SPTB-LTERM	0.035
Lactate (1.33)	1	0.004	SPTB-LTERM	0.043
			PPROM-LTERM	0.044
Lactate (4.11)	1	0.004	PPROM-LTERM	0.038
			SPTB-LTERM	0.048
Unknown (4.40)	4	0.005	PPROM-LTERM	0.026
Propylene-glycol (1.15)	1	0.005	PPROM-LTERM	0.002
			PPROM-HTERM	0.017
Mobile lipids (1.23)	3	0.01	LTERM-HTERM	0.010
Choline (3.19)	2	0.011	LTERM-HTERM	0.026
2-Hydroxyvalerate (4.07)	2	0.012	SPTB-LTERM	0.030
Proline (2.33)	1	0.012	SPTB-LTERM	0.046
NDMA (3.15)	2	0.013	LTERM-HTERM	0.018
3-Hydroxybutyrate (4.16)	2	0.013	SPTB-LTERM	0.014
Myoinositol (3.58)	1	0.013	SPTB-LTERM	0.027
3-Hydroxybutyrate (1.20)	2	0.015	LTERM-HTERM	0.036
Glucarate (4.14)	2	0.016	SPTB-LTERM	0.022
Acetoacetate (2.23)	1	0.017	SPTB-LTERM	0.043
Unknown (1.09)	4	0.017	LTERM-HTERM	0.047
Glutamate (2.26)	1	0.018	NA	NA
2-Hydroxyvalerate and arginine (1.62)	2 and 1	0.02	NA	NA
Unknown (3.34)	4	0.021	NA	NA
Mobile lipids (1.29)	3	0.03	NA	NA
Unknown (1.41)	4	0.031	NA	NA
Unknown (4.46)	4	0.032	NA	NA
Unknown (1.54)	4	0.032	NA	NA
3-Hydroxybutyrate (2.42)	2	0.039	SPTB-LTERM	0.025
Mannose (5.19)	1	0.046	NA	NA
Glutamate (2.48)	1	0.047	LTERM-HTERM	0.035
Phenylalanine (7.43)	1	0.049	NA	NA
Unknown (2.78)	4	0.049	NA	NA

Of these bins, 24 were annotated metabolites and 10 were unknown [28]. HTERM (*n*=108), LTERM (*n*=172), PPROM (*n*=16) and SPTB (*n*=22).

NA = the individual outcome comparison is borderline *P* > 0.05 and therefore does not meet the *P* ≤ 0.05 cut-off.

Multivariate supervised discriminant analysis demonstrated no clear separation of clusters of the four different clinical groups and poor prediction. At week 16, PLS-DA yielded an *R*^2^ = 0.034, *Q*^2^ = 0.014, 3-components (Supplementary Figure S2). Similarly, at week 20, PLS-DA model yielded *R*^2^ = 0.06, *Q*^2^ = 0.007, 3-components (Supplementary Figure S3).

### Predictive modelling using probabilistic neural networks

**Figure 2:**
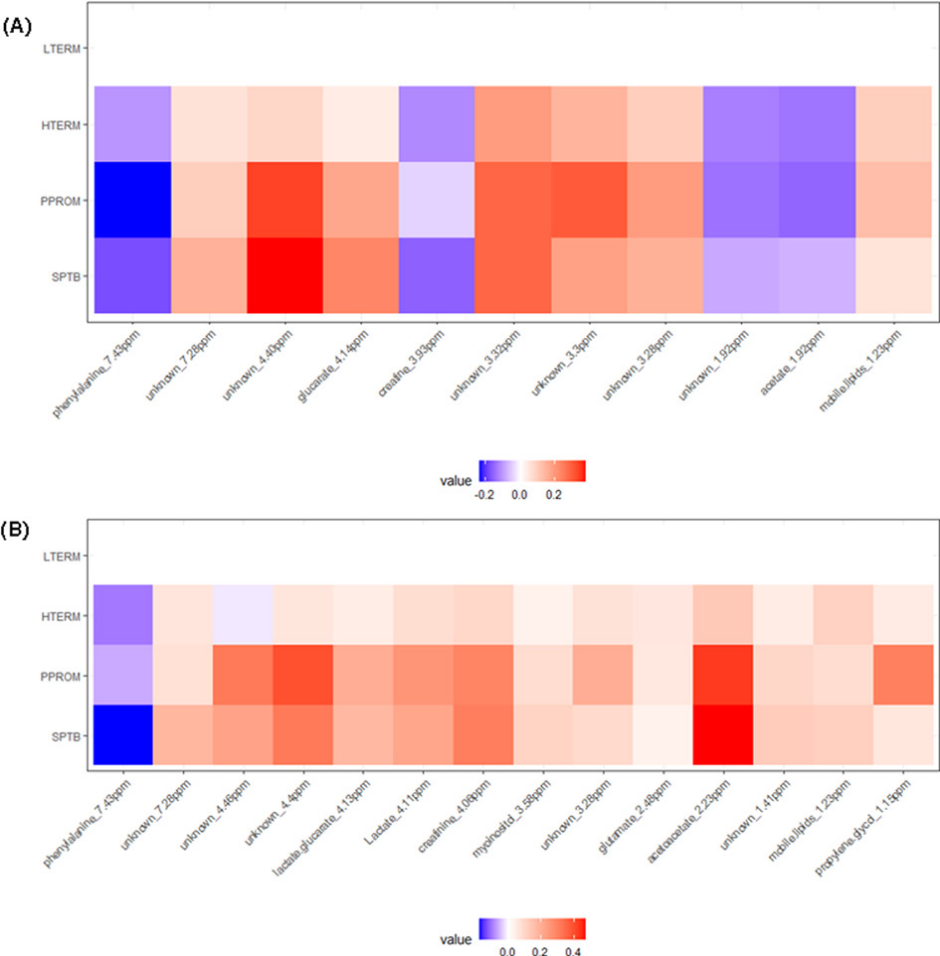
Log fold change diagrams of ^1^H NMR significant metabolite bins identified from univariate and multivariate analyses Individual pregnancy outcome groups were compared, where LTERM was the control group at (**A**) week 16 of gestation (11 metabolite bins) (*N*=329) and (**B**) week 20 of gestation (14 metabolite bins) (*N*=318). Red indicates positive fold change and blue for negative fold change with respect to LTERM. Plots were generated using ‘ggplot2’ R package [51] and R script developed by R. Grosman, 2017 (University of Liverpool).

As PLS-DA did not show clear discrimination between SPTB and PPROM, PNN analysis of all the metabolite bins was conducted between sPTB cases combined (PPROM and SPTB) and controls at week 16 gestation and showed moderate predictive power AUC = 0.77 (LOOCV) (Supplementary Table S1). Unknown (3.32 ppm) bin had the highest rank in the week 16 prediction model, which is consistent with the univariate analyses as shown in the log fold change diagram [51] (Tables 2 and 3; Figure 2A).

PNN analysis at week 20 of gestation obtained stronger predictive power with AUC = 0.89 than week 16 (Supplementary Table S1). Unknown (7.28 ppm) was another top hit followed by creatinine (4.06 ppm) as with the ANOVA results. Further matches with ANOVA at week 20 were observed for lactate and glucarate (4.13 ppm), lactate (4.11 ppm) and phenylalanine (7.43 ppm), which also scored highly in the PNN model (Figure 2B).

### MGWAS and SNP annotation results

**Figure 3:**
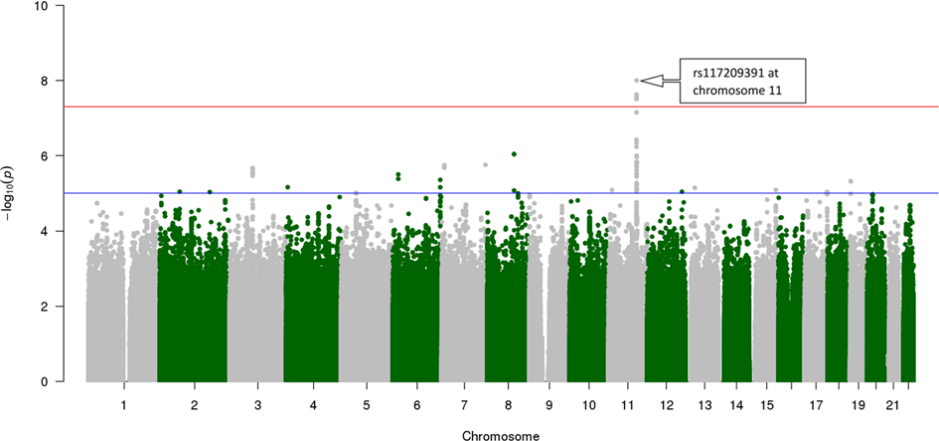
Manhattan plot of phenylalanine (7.43 ppm) metabolite bin MGWAS analysis at week 16 of gestation Spectra were obtained from ^1^H NMR of preterm birth maternal serum (*N*=251). Chromosome 11 SNP rs117209391 (*P* = 9.96 × 10^−9^), reached genome-wide significance (*P* < 5 × 10^−8^, red line). A strong association was observed between phenylalanine (7.43 ppm) metabolite peak intensity and genome-wide data. This plot was generated using R package, qqman [47].

A total of 251 women at week 16 and 265 women at week 20 had both GWAS and NMR data available and were included in MGWAS analyses (Figure 1C). Of 290 MGWAS analyses at both timepoints, the lowest *P*-value and strongest genetic association with relative peak intensity was observed at week 16 for phenylalanine (7.43 ppm) with rs117209391 (a non-coding RNA, see Supplementary Table S2) reaching genome-wide significance (Figure 3). Several signals observed across the remaining MGWAS analyses did not reach the genome-wide significance threshold.

Genome-wide significant (*P* < 5 × 10^−8^) SNPs in nine metabolite bins were identified, including phenylalanine (7.43 ppm), 2-hydroxybutyrate (0.92 ppm), proline (3.37 ppm), lactate (4.11 ppm), unknown (7.06 ppm) and proline (2.33 ppm) at week 16 and glucose (3.77 ppm), myoinositol (3.58 ppm) and lactate (4.11 ppm) at week 20 (Supplementary Table S2).

FUMA SNP annotation of MGWAS results identified one exonic SNP in unknown (5.39 ppm), week 16 metabolite bin (chr:bp 20:19941367, rs45481396, *P* = 1.85E-06), the remaining SNPs were in non-coding regions. Rs45481396, located in a protein coding gene *RIN2* (Ras and Rab Interactor 2), is involved in membrane trafficking processes.

### eQTL and enrichment analysis

*Cis*-eQTL mapping of genome-wide significant SNPs identified one gene, *TRAF1* (tumour necrosis factor receptor associated factor 1) for lactate (4.11 ppm) at week 16 (Supplementary Table S2). This suggests that the non-coding SNP detected in the week 16 lactate (4.11 ppm) MGWAS (rs7867041, intergenic RP11-360A18.1, *P* = 3.08 × 10^−8^) could influence gene expression of the neighbouring gene, *TRAF1*. *TRAF1* is involved in multiple immune/inflammatory related pathways such as TNF signalling and NF-kappa B signalling pathway (including interleukins) (KEGG ID: 04064) as recorded in the KEGG database [52].

However, gene set enrichment analysis with the GTEx database did not show any differential expression in reproductive or pregnancy-associated tissues. Contrary to this, enrichment analysis of lactate (4.11 ppm) at week 20, demonstrated that gene sets were upregulated in breast tissue (FDR, *P* = 0.039) in GTEx (Supplementary Figure S4). This was also true for uterus (*P* = 0.032), but this was not significant after FDR adjustment (FDR, *P* = 0.97).

## Discussion

Significant differences (*P* < 0.05) determined between term and sPTB in the metabolite profiles include phenylalanine (7.43 ppm) and acetate (1.92 ppm) at week 16 and creatinine (4.06 ppm), lactate and glucarate (4.13 ppm), lactate (4.11 ppm) and lactate (1.33 ppm) at week 20 between controls and sPTB cases (Tables 1 and 2; Figure 2). PNN also showed strong contribution of these metabolites, particularly at week 20 of gestation (AUC = 0.89).

Acetate and lactate were detected in cervicovaginal fluid, using NMR, in preterm symptomatic pregnant women at 20–22 weeks of gestation [6]. Phenylalanine (and leucine/isoleucine, histidine, and valine) were found in amniotic fluid, at 16–21 weeks gestation, investigated using mass-spectrometry [12]. Our cohort, comprised of sPTB cases and healthy controls at two early gestational timepoints, yielded similar results as shown by ANOVA and PNN.

There are several strengths of our study: (1) clinically well-defined phenotype in early pregnancy of a prospective cohort of women with pre-defined inclusion/exclusion criteria and clearly defined control groups: first, women at high-risk of sPTB with a history of sPTB in previous pregnancy; second, women at low-risk of sPTB with previous term birth; (2) inclusion of sPTB cases only if ≤ 34 weeks at birth; (3) availability of multi-omic data in the same individual at two timepoints and the novelty in analytical approaches including PNN; (4) rigorous quality control of clinical, experimental and analytical data.

One novel aspect of the present study was the combination of metabolomics and genotype data from the same participants in the sPTB cohort, which were applied in MGWAS association analyses. Identification of SNP associations with each metabolite bin was a non-standard approach implemented to gain insights of molecular pathways contributing to sPTB phenotypes. A strong genetic association of phenylalanine (7.43 ppm) and lactate (4.11 ppm) bins at week 16 and lactate (4.11 ppm) at week 20 with the sPTB cohort genome-wide data were determined (Figure 3). Due to low prevalence of these SNPs in this cohort, the outcomes could not be associated with the SNPs; however, meta-analysis with similar cohorts could allow for a robust comparison.

SNP annotation of the known bins indicated non-coding gene regions. FUMA cis-eQTL analysis identified neighbouring gene, *TRAF1*, in association with the leading SNP significant (*P* < 5 × 10^−8^) in lactate (4.11 ppm) metabolite bin at week 16 of gestation. *TRAF1* is related to similar signalling pathways involving NF-κβ, of which *NFKB1* gene was previously reported in a literature-informed analysis by Bacelis et al. [53] and reviewed by MacIntyre et al. [54] as a potential biomarker of PTB. TRAF1 forms a heterodimeric complex with TRAF2, which was associated with PTB [55]. The complex acts as a mediator of anti-apoptotic signals from TNF receptors [55], which also indicates the role of the *TRAF1* gene in the inflammatory pathway. Other studies have indicated the role of innate immune cells in producing lactate when activated by an inflammation response [56,57]. This suggests that *TRAF1* could be part of the activation of inflammation processes, which in turn activate immune cells that secretes lactate detectable in serum. This could explain the lactate differences between sPTB cases and controls identified by ANOVA and PNN. Further analysis is required to confirm this hypothesis, however, raised lactate indicating disease other than a non-genetic biological response (e.g. sepsis and cardiac arrest [58]) is highly unlikely as these women presented well to their outpatient clinical appointments and were reviewed by obstetricians trained in the identification of disease in pregnancy.

Gene set enrichment analysis with GTEx database highlighted lactate at week 20 elevated in cervix tissue (Supplementary Figure S4) has been previously reported in the literature [6]. Our enrichment analysis suggested that lactate was elevated in breast tissue, but there is no association with preterm birth in the current literature.

Some limitations of the study include the variability in metabolic profiles in NMR studies on biofluids caused by exogenous substances or environmental factors, such as drugs or food intake. This was taken into consideration in the present study by following up the univariate analysis with PNN. This unbiased modelling approach confirmed contribution of similar metabolites as found in the ANOVA.

Despite reporting on one of the largest prospective recruitments of sPTB < 34 weeks cases by targeting a high-risk population, the number of overall cases were low especially when sub-classified into SPTB and PPROM. However, the study design allowed univariate and multivariate analyses at two timepoints and with multiple omics datasets in this phenotypically well-defined cohort. ANOVA identified many differences between LTERM (healthy controls) and sPTB cases, which could contribute to the preterm phenotypes. However, the metabolic profile of HTERM women could share similarities with those who experience a recurrent sPTB, as all high-risk participants previously experienced sPTB, unlike LTERM. PLS-DA could not distinguish between the four phenotype groups, potentially due to the low numbers; however, combining sPTB cases and controls for PNN analysis demonstrated differences between cases and controls, whilst displaying predictive power.

There are several unknown metabolites associated with ANOVA and PNN. Further work is underway to attempt to identify these unknown metabolites. Proteomics data for this cohort are presently being gathered. Analysis of this data will provide a mechanistic insight of how the genes identified may influence the production of metabolites, which could influence the pathophysiology of PTB.

To our knowledge, this is the first study to have used NMR metabolic profiling of a clearly defined cohort of high and low-risk sPTB participants. Another unique aspect was the collection of multiple omics data from the same participants, which highlighted the potential role of several metabolites in the initiation of early labour. This phenotypically well-defined cohort of patient samples provides invaluable resources for future studies to be undertaken to enhance our understanding of the sPTB pathophysiology.

## Clinical perspectives

- Spontaneous preterm births (PTB) are a major cause of infant morbidity and mortality worldwide. This condition remains poorly understood and there is a need for biomarkers that can aid patient stratification and predict phenotypes of spontaneous preterm birth.
- The present study demonstrated that metabolite signatures differ between spontaneous PTB cases and term controls. Further analysis of metabolomic data with genomic data from the same participants indicate the potential role of the inflammation pathway via the *TRAF1* gene.
- This is the first study to recruit and phenotype pregnant women for multi-omic investigation at high-risk and low-risk of spontaneous preterm birth. Further validation of these multi-omic biomarkers may allow screening for spontaneous preterm birth risk in asymptomatic women in early pregnancy.

## Supplementary Material

Supplementary Figures S1-S4 and Tables S1-S2

## Data Availability

Metabolomic analysis data are available in the open-access repository MetaboLights, study ID: MTBLS1990.

## Open Access

Open access for this article was enabled by the participation of University of Liverpool in an all-inclusive *Read & Publish* pilot with Portland Press and the Biochemical Society under a transformative agreement with JISC.

## Abbreviations

ANOVA: analysis of variance
AUC: area under the curve
EQTL: expression quantitative trait loci
GTEx: Genotype-Tissue Expression Project
HTERM: high-risk term
LLETZ: large loop excision of the transformation zone
LOOCV: leave-one-out cross validation
LTERM: low-risk term
MGWAS: metabolomics genome-wide association
NMR: nuclear magnetic resonance
PNN: probabilistic neural network
PPROM: preterm premature rupture of membranes
PTB: preterm birth
SNP: single-nucleotide polymorphism
SPTB: spontaneous preterm birth
