# Supplementary material for: Metabolic profiling of maternal serum of women at high-risk of spontaneous preterm birth using NMR and MGWAS approach
Source: Biosci Rep. 2021 Aug 31;41(9):BSR20210759. doi: 10.1042/BSR20210759 (PMC8415214; doi:10.1042/BSR20210759)
Supplement: Supplementary Figures S1-S4 and Tables S1-S2 [file BSR-2021-0759_supp.pdf]

Supplementary Information

Metabolic profiling of maternal serum of women at high-risk of spontaneous preterm birth using NMR and mGWAS approach

Juhi K. Gupta[1,2]***, *Angharad Care*[2], *Laura Goodfellow*[2], *Zarko Alfirevic*[2], *Lu-Yun Lian*[4], *Bertram Müller-Myhsok*[1, 3], *Ana Alfirevic*[1,2], *Marie M. Phelan*[4]

[1] Wolfson Centre for Personalised Medicine, Department of Pharmacology and Therapeutics, Institute of Systems, Molecular and Integrative Biology, University of Liverpool, Liverpool, L69 3GL, UK

[2] Harris-Wellbeing Research Centre, University Department, Liverpool Women's Hospital, Crown Street, Liverpool, L8 7SS, UK

[3]Max Planck Institute of Psychiatry, 80804, Munich, Germany

[4]NMR Centre for Structural Biology, Institute of Systems, Molecular and Integrative Biology, University of Liverpool, Liverpool, L69 7ZB, UK

Table of contents

Figure S1. Twenty-four representative serum NMR spectra with metabolite standards from the PTB cohort.

Figure S2. PLS-DA of metabolite bins at week 16 of gestation.

Figure S3. PLS-DA of metabolite bins at week 20 of gestation.

Table S1. Probabilistic neural network (PNN) results at week 16 and 20 of gestation.

Table S2. FUMA SNP annotation and gene set enrichment analysis of significant SNPs from mGWAS.

Figure S4. Enriched differentially expressed gene sets (DEG) from lactate (4.11 ppm) mGWAS analysis at week 20 of gestation.

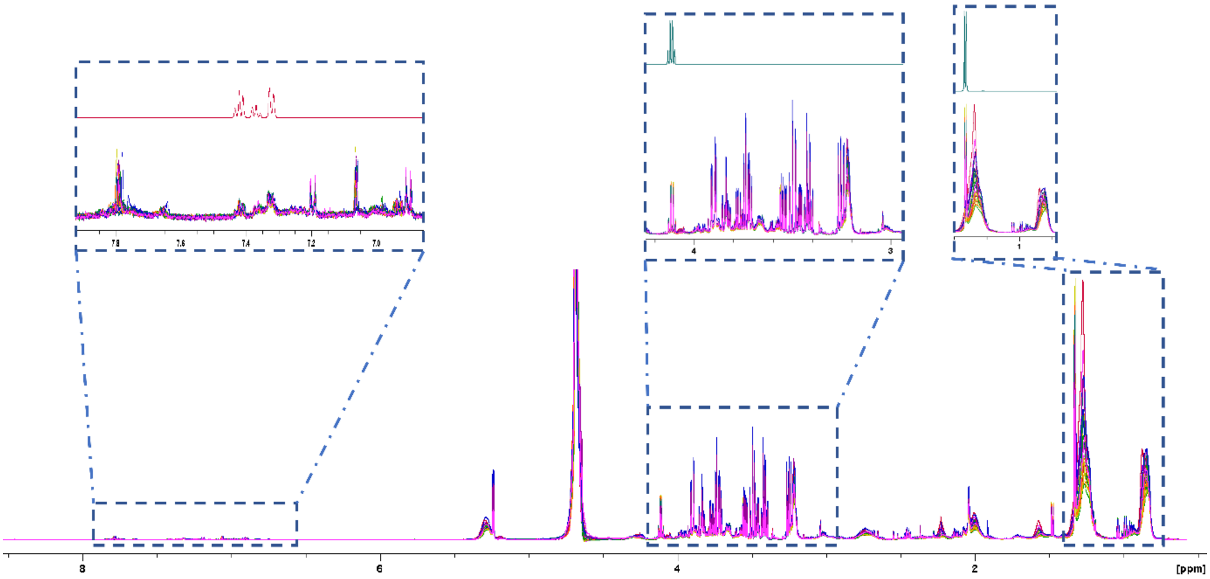

Figure S1. **Twenty-four representative serum NMR spectra with metabolite standards from the PTB cohort**. The top 3 inserts highlight the regions of interest from the spectra and their corresponding metabolite standards. From left to right, region 7.9-6.9 ppm shows stacked plot with phenylalanine aromatic peaks from in-house standard (red spectrum), region 4.2-3.0 ppm shows stacked plot with lactate quartet peak from in-house standard (green spectrum) and region 1.4-0.8 ppm shows stacked plot with lactate doublet peak from in-house standard (green spectrum).

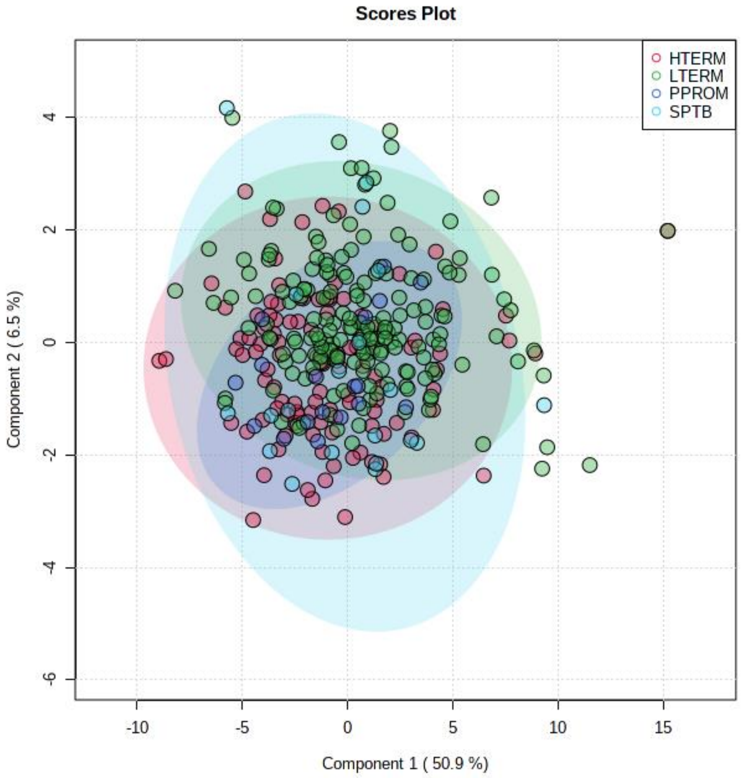

Figure S2. PLS-DA score plot of metabolite bins at week 16 of gestation. Variance explained is shown by the percentage for each component. This figure generated using MetaboAnalyst [34].

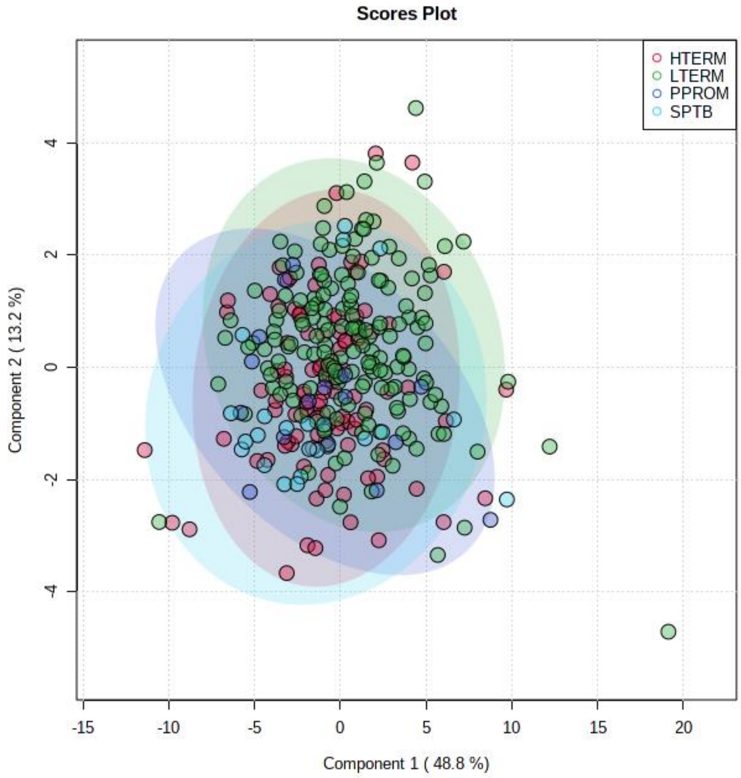

Figure S3. PLS-DA score plot of metabolite bins at week 20 of gestation. Variance explained is shown by the percentage for each component. This figure generated using MetaboAnalyst [34].

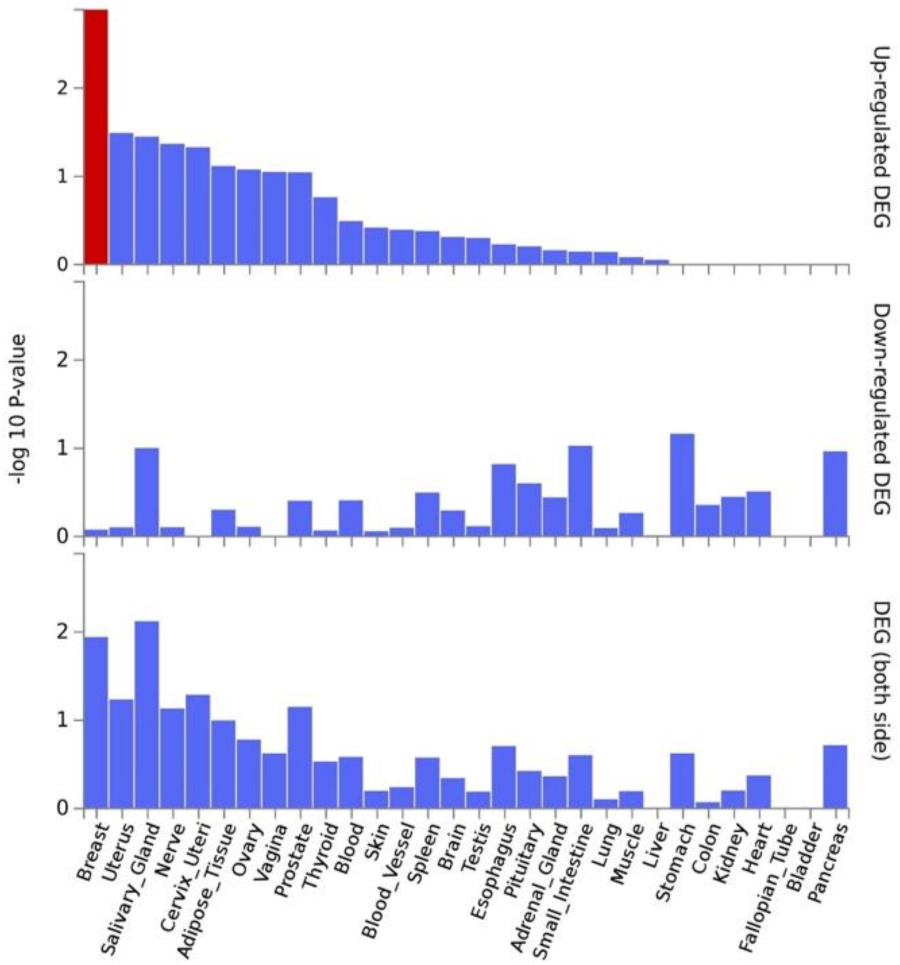

Figure S4. **Enriched differentially expressed gene sets (DEG) from lactate (4.11 ppm) mGWAS analysis at week 20 of gestation.** DEGs were defined by two-sided t-tests applied across the mGWAS genes and tissue types from the GTEx v8 database in FUMA GWAS software [48]. DEG in breast tissue (red) was significantly enriched (Bonferroni p<0.05) followed by uterus tissue (also upregulated).

Table S1. Probabilistic neural network (PNN) modelling of metabolite bins at week 16 and 20 of gestation. Week 20 showed stronger prediction (AUC, C-statistics=0.887). At week 16 of gestation the AUC value (or C-statsitic) achieved was 0.766.

**Week 16 Validation Data**		**Week 20 Validation Data**	
Actual : ----Predicted Category---<br>Category: case control<br>--------: ------------ ------------<br>case: 12 26<br>control: 1 290<br><br>-------- Validation Data --------<br><br>Total records = 329<br>Positive/Negative ratio = 7.6579<br>Accuracy = 91.79%<br>True positive (TP) = 290 (88.15%)<br>True negative (TN) = 12 (3.65%)<br>False positive (FP) = 26 (7.90%)<br>False negative (FN) = 1 (0.30%)<br>Sensitivity = 99.66%<br>Specificity = 31.58%<br>Geometric mean of sensitivity and specificity = 56.10%<br>Positive Predictive Value (PPV) = 91.77%<br>Negative Predictive Value (NPV) = 92.31%<br>Geometric mean of PPV and NPV = 92.04%<br>Precision = 91.77%<br>Recall = 99.66%<br>F-Measure = 0.9555<br>Area under ROC curve (AUC, C-Statistic) = 0.766323		Actual : ----Predicted Category---<br>Category: case control<br>--------: ------------ ------------<br>case: 30 8<br>control: 0 280<br><br>-------- Validation Data --------<br><br>Total records = 318<br>Positive/Negative ratio = 0.1357<br>Accuracy = 97.48%<br>True positive (TP) = 30 (9.43%)<br>True negative (TN) = 280 (88.05%)<br>False positive (FP) = 0 (0.00%)<br>False negative (FN) = 8 (2.52%)<br>Sensitivity = 78.95%<br>Specificity = 100.00%<br>Geometric mean of sensitivity and specificity = 88.85%<br>Positive Predictive Value (PPV) = 100.00%<br>Negative Predictive Value (NPV) = 97.22%<br>Geometric mean of PPV and NPV = 98.60%<br>Precision = 100.00%<br>Recall = 78.95%<br>F-Measure = 0.8824<br>Area under ROC curve (AUC, C-Statistic) = 0.886842	
**Week 16 metabolite bin (chemical shift in ppm)**	**Importance score**	**Week 20 metabolite bin (chemical shift in ppm)**	**Importance score**
unknown (3.32)	100	unknown (7.28)	100
proline (3.37)	95.582	phenylalanine (7.34)	61.121
phenylalanine (7.43)	87.738	creatinine (4.06)	59.146
unknown (7.28)	79.361	desaminotyrosine (7.15)	31.221
unknown (4.4)	0.776	glucose (3.41)	25.793
glucarate (4.14)	0.648	phenylalanine (7.43)	17.521
3-hydroxybutyrate (1.2)	0.47	acetate (1.92)	17.317
desaminotyrosine (6.83)	0.454	unknown (3.3)	14.371
unknown (2.7)	0.454	desaminotyrosine (6.83)	11.222
unknown (2.68)	0.432	unknown (3.64)	10.373
unknown (4.46)	0.423	unknown (3.64)	7.792
desaminotyrosine (7.15)	0.421	histidine (7.81)	7.062
citrate (2.67)	0.391	acetoacetate (2.23)	7.001
mobile-lipids & 2-hydroxyisovalerate (0.84)	0.365	lactate/glucarate (4.13)	4.071
citrate (2.7)	0.364	glucose (3.4)	3.743
unknown (2.72)	0.363	unknown (4.4)	3.217
glucose (3.24)	0.362	glucose (3.46)	2.191
citrate (2.75)	0.35	glucose/unknown (3.71)	2.138
2-hydroxybutyrate (0.92)	0.348	unknown (3.67)	1.393
unknown (8.2)	0.331	myoinositol (3.58)	1.393

isopropanol (1.18)	0.329	proline (3.37)	0.765
unknown (2.74)	0.323	glucose (3.54)	0.725
unknown (7.38)	0.295	glucose (3.41)	0.7
mobile lipids (5.31)	0.291	glucose (3.44)	0.694
mobile lipids (1.23)	0.285	propylene-glycol (1.15)	0.688
2-hydroxyvalerate (4.07)	0.284	glucose (3.72)	0.647
leucine (0.94)	0.28	glucose (3.9)	0.614
unknown (3.34)	0.265	glucose (3.5)	0.599
lysine (3.05)	0.262	glucose (3.47)	0.574
histidine/unknown (3.17)	0.262	glucose (3.82)	0.561
unknown (2.78)	0.258	glucose (3.48)	0.551
mobile lipids (1.96)	0.257	glucose (3.49)	0.536
glutamate (2.26)	0.256	glucose (3.43)	0.525
mobile lipids (0.9)	0.252	glucose (3.27)	0.522
unknown (1.09)	0.239	glucose (3.25)	0.518
creatinine (4.06)	0.238	histidine/unknown (3.17)	0.514
glucarate/myoinositol (4.04)	0.226	glucose (3.42)	0.51
unknown (3.28)	0.219	glucose (3.74)	0.51
unknown (4.32)	0.218	glucose (3.55)	0.5
3-hydroxybutyrate (4.16)	0.212	glucose (3.24)	0.461
unknown (3.64)	0.202	glucose (5.24)	0.451
unknown (3.64)	0.199	glutamine (2.14)	0.447
unknown (7.06)	0.197	glucose (3.84)	0.436
histidine (7.06)	0.196	unknown (3.81)	0.362
unknown (2.89)	0.192	glucose (3.86)	0.356
proline (2.33)	0.192	glucose (3.79)	0.326
histidine & unknown (3.13)	0.184	unknown (3.26)	0.303
unknown (2.83)	0.184	glucose (3.52)	0.3
glutamate (2.48)	0.181	unknown (3)	0.289
arginine & phosophocholine (3.22)	0.178	glucose (3.78)	0.259
proline & glutamate (2.01)	0.177	glucose (3.91)	0.251
3-hydroxybutyrate (2.42)	0.174	glucose (3.75)	0.245
unknown (1.41)	0.172	unknown (3.28)	0.228
myoinositol (3.58)	0.172	unknown (3.76)	0.223
unknown (1.86)	0.167	unknown (4.46)	0.202
propylene-glycol (1.15)	0.161	unknown (5.39)	0.171
lysine (3.03)	0.161	mobile lipids (1.23)	0.133
unknown (1.37)	0.157	mobile lipids (1.96)	0.123
mobile lipids (1.29)	0.153	unknown (2.89)	0.118
glycylproline (2.05)	0.147	tyrosine (7.2)	0.087
Isoleucine/Leucine (0.95)	0.137	unknown (3.63)	0.081
unknown (1.45)	0.136	glutamate (2.48)	0.063
unknown (1.54)	0.125	unknown (7)	0.059
unknown (1.93)	0.123	lactate (4.11)	0.055
phenylalanine (7.34)	0.11	unknown (7.23)	0.049
glutamate/proline (2.09)	0.106	unknown (1.41)	0.048
unknown (3.67)	0.102	histidine/unknown (3.13)	0.047
unknown (5.39)	0.095	unknown (2.7)	0.043
arginine (1.89)	0.09	unknown (2.44)	0.033
creatinine (3.04)	0.088	unknown (3.88)	0.004
acetoacetate (2.23)	0.087	unknown (2.58)	0.003
unknown (3.76)	0.084	creatinine (3.04)	0.002
unknown (3.81)	0.075		
unknown (7.33)	0.072		

glutamine (2.14)	0.067		
phenylalanine (7.31)	0.067		
Isoleucine (1.02)	0.065		
unknown (2.58)	0.054		
mannose (5.19)	0.051		
Isoleucine (0.97)	0.044		
glutamate & 3-hydroxybutyrate (2.27)	0.042		
unknown (3.35)	0.039		
histidine (7.81)	0.039		
lipid & unknown (3.97)	0.032		
creatine (3.93)	0.026		
acetate (1.92)	0.022		
valine (0.99)	0.015		
lactate (1.33)	0.012		
valine (1.04)	0.011		
unknown (3.3)	0.009		
unknown (2.44)	0.008		
unknown (1.92)	0.005		

Table S2. FUMA SNP annotation and gene set enrichment analysis of significant SNPs from mGWAS. Nine genome-wide significant ($p<5x10^{-8}$) SNPs from mGWAS analysis metabolite bins were obtained. SNPs *cis*-eQTL were mapped to GTex v8 tissue database.
NA = No significant eQTL or tissue identified.
* = upregulated differentially expressed gene sets, significant after FDR (p<0.05)

	Metabolite bin (chemical shift in ppm)	Chr	rsID	P	Gene	Function	cis-eQTL	Tissue
Week 16	phenylalanine (7.43)	11:97799649	rs117209391	9.96E-09	RP11-379J13.2	ncRNA intronic	NA	NA
	2-hydroxybutyrate (0.92)	13:107518761	rs9301166	1.36E-08	PPIAP24	intergenic	NA	NA
	proline (3.37)	6:94722833	rs116984633	2.84E-08	RP11-524K14.1	intergenic	NA	NA
	**lactate (4.11)**	9:122774973	rs7867041	3.08E-08	RP11-360A18.1	intergenic	TRAF1	NA
	unknown (7.06)	15:27961561	rs3097466	3.56E-08	RP11-30G8.1	intergenic	NA	NA
	proline (2.33)	18:44144080	rs117635196	3.63E-08	LOXHD1	intronic	NA	NA
Week 20	glucose (3.77)	2:169351131	rs11897514	2.58E-08	CERS6	intronic	NA	NA
	myoinositol (3.58)	13:112191253	rs9522264	2.88E-08	RP11-65D24.2	intergenic	NA	NA
	lactate (4.11)	4:30111285	rs79350708	3.53E-08	RP11-174E22.2	intergenic	NA	Breast*
